# Wall shear stress measured with 4D flow CMR correlates with biomarkers of inflammation and collagen synthesis in mild-to-moderate ascending aortic dilation and tricuspid aortic valves

**DOI:** 10.1093/ehjci/jeae130

**Published:** 2024-05-15

**Authors:** Filip Hammaréus, Chiara Trenti, Hanna M Björck, Jan Engvall, Hanna Lekedal, Aleksandra Krzynska-Trzebiatowska, David Kylhammar, Marcus Lindenberger, Anna K Lundberg, Fredrik Nilsson, Lennart Nilsson, Eva Swahn, Lena Jonasson, Petter Dyverfeldt

**Affiliations:** Department of Internal Medicine in Jönköping, and Department of Health, Medicine and Caring Sciences, Linköping University, Länssjukhuset Ryhov, Sjukhusgatan, 551 85 Jönköping, Sweden; Division of Diagnostics and Specialist Medicine, Department of Health, Medicine and Caring Sciences, Linköping University, Universitetssjukhuset, 581 83 Linköping, Sweden; Division of Diagnostics and Specialist Medicine, Department of Health, Medicine and Caring Sciences, Linköping University, Universitetssjukhuset, 581 83 Linköping, Sweden; Center for Medical Image Science and Visualization, Linköping University, Universitetssjukhuset, 581 83 Linköping, Sweden; Division of Cardiovascular Medicine, Center for Molecular Medicine, Department of Medicine, Karolinska Institute, Karolinska University Hospital, Visionsgatan 18, Stockholm, 171 76 Solna, Sweden; Division of Diagnostics and Specialist Medicine, Department of Health, Medicine and Caring Sciences, Linköping University, Universitetssjukhuset, 581 83 Linköping, Sweden; Department of Clinical Physiology and Department of Health, Medicine and Caring Sciences, Linköping University, Universitetssjukhuset, 581 83 Linköping, Sweden; Östersund Hospital, Östersundssjukhus, 831 83 Östersund, Sweden; Department of Cardiology and Department of Health, Medicine and Caring Sciences, Linköping University, Universitetssjukhuset, 581 83 Linköping, Sweden; Department of Clinical Physiology and Department of Health, Medicine and Caring Sciences, Linköping University, Universitetssjukhuset, 581 83 Linköping, Sweden; Wallenberg Centre for Molecular Medicine, Linköping University, Universitetssjukhuset, 581 83 Linköping, Sweden; Department of Cardiology and Department of Health, Medicine and Caring Sciences, Linköping University, Universitetssjukhuset, 581 83 Linköping, Sweden; Division of Diagnostics and Specialist Medicine, Department of Health, Medicine and Caring Sciences, Linköping University, Universitetssjukhuset, 581 83 Linköping, Sweden; Department of Cardiology and Department of Health, Medicine and Caring Sciences, Linköping University, Universitetssjukhuset, 581 83 Linköping, Sweden; Department of Internal Medicine in Jönköping, and Department of Health, Medicine and Caring Sciences, Linköping University, Länssjukhuset Ryhov, Sjukhusgatan, 551 85 Jönköping, Sweden; Division of Diagnostics and Specialist Medicine, Department of Health, Medicine and Caring Sciences, Linköping University, Universitetssjukhuset, 581 83 Linköping, Sweden; Department of Internal Medicine in Jönköping, and Department of Health, Medicine and Caring Sciences, Linköping University, Länssjukhuset Ryhov, Sjukhusgatan, 551 85 Jönköping, Sweden; Department of Cardiology and Department of Health, Medicine and Caring Sciences, Linköping University, Universitetssjukhuset, 581 83 Linköping, Sweden; Division of Diagnostics and Specialist Medicine, Department of Health, Medicine and Caring Sciences, Linköping University, Universitetssjukhuset, 581 83 Linköping, Sweden; Department of Cardiology and Department of Health, Medicine and Caring Sciences, Linköping University, Universitetssjukhuset, 581 83 Linköping, Sweden; Division of Diagnostics and Specialist Medicine, Department of Health, Medicine and Caring Sciences, Linköping University, Universitetssjukhuset, 581 83 Linköping, Sweden; Center for Medical Image Science and Visualization, Linköping University, Universitetssjukhuset, 581 83 Linköping, Sweden

**Keywords:** aortic dilation, wall shear stress, circulating biomarkers, cardiovascular magnetic resonance, 4D flow CMR

## Abstract

**Aims:**

Understanding the mechanisms underlying ascending aortic dilation is imperative for refined risk stratification of these patients, particularly among incidentally identified patients, most commonly presenting with tricuspid valves. The aim of this study was to explore associations between ascending aortic haemodynamics, assessed using four-dimensional flow cardiovascular magnetic resonance imaging (4D flow CMR), and circulating biomarkers in aortic dilation.

**Methods and results:**

Forty-seven cases with aortic dilation (diameter ≥ 40 mm) and 50 sex-and age-matched controls (diameter < 40 mm), all with tricuspid aortic valves, underwent 4D flow CMR and venous blood sampling. Associations between flow displacement, wall shear stress (WSS), and oscillatory shear index in the ascending aorta derived from 4D flow CMR, and biomarkers including interleukin-6, collagen type I α1 chain, metalloproteinases (MMPs), and inhibitors of MMPs derived from blood plasma, were investigated. Cases with dilation exhibited lower peak systolic WSS, higher flow displacement, and higher mean oscillatory shear index compared with controls without dilation. No significant differences in biomarkers were observed between the groups. Correlations between haemodynamics and biomarkers were observed, particularly between maximum time-averaged WSS and interleukin-6 (*r* = 0.539, *P* < 0.001), and maximum oscillatory shear index and collagen type I α1 chain (*r* = −0.575, *P* < 0.001 in cases).

**Conclusion:**

Significant associations were discovered between 4D flow CMR derived whole-cardiac cycle WSS and circulating biomarkers representing inflammation and collagen synthesis, suggesting an intricate interplay between haemodynamics and the processes of inflammation and collagen synthesis in patients with early aortic dilation and tricuspid aortic valves.

## Graphical Abstract

**Graphical Abstract:**
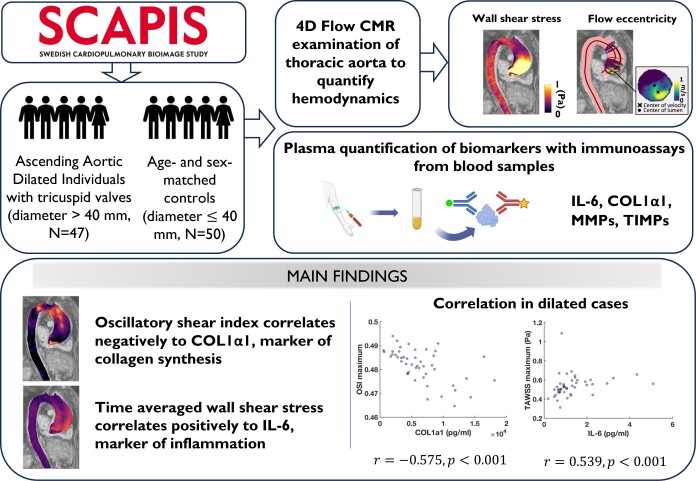
Individuals with tricuspid aortic valves and ascending aortic dilation (diameter ≥ 40 mm, *n* = 47) and sex- and age-matched controls (diameter < 40 mm, *n* = 50) were recruited from the SCAPIS study and underwent a 4D flow CMR examination to quantify wall shear stress and flow eccentricity, while plasma biomarkers were quantified with immunoassays, showing that biomarkers of collagen synthesis and inflammation correlated with altered ascending aortic haemodynamics. 4D flow CMR, four-dimensional flow cardiac magnetic resonance imaging; IL-6, interleukin-6; COL1α1, type I collagen α1 chain; MMP, matrix metalloproteinase; TIMP, tissue inhibitor of MMP; WSS, wall shear stress; TAWSS, time-averaged WSS; OSI, oscillatory shear index; TAV, tricuspid aortic valve.

## Introduction

Aortic dilation typically manifests as an asymptomatic condition, which is detected incidentally or when symptoms arise from aortic regurgitation, dissection, or rupture.^1^ The number of incidentally detected patients with aortic dilation is increasing because of the more frequent use of thoracic imaging in the diagnosis of coexisting cardiovascular and pulmonary diseases. Current guidelines for these patients suggest regular surveillance imaging until the ascending aortic diameter reaches a threshold for prophylactic surgery.^2^ However, most aortas grow slowly and only a minority of patients suffer complications.^1,3^ Further, most dissections of the ascending aorta occur when the diameter is below the threshold for surgery.^4^ An improved understanding of the pathophysiology of aortic dilation and risk for disease progression with ensuing catastrophic events is hence needed to optimize follow-up strategies and decisions for prophylactic surgery.

Mounting evidence supports the notion that altered haemodynamics measured with three-dimensional cine phase-contrast cardiovascular magnetic resonance (4D flow CMR) could improve risk stratification of aortic dilation.^5–8^ For example, elevated regional wall shear stress (WSS) has been associated with aortic growth rate in patients with bicuspid aortic valves (BAV).^5,6^ Further, aortic dilation and growth rate have been associated with overall low WSS in individuals with tricuspid aortic valves (TAVs) and no aortic stenosis.^8,9^ Moreover, eccentric flow in the proximal ascending aorta has been found in patients with aortic dilation and higher growth rates.^8,10^ The role of altered haemodynamics in aortic dilation has largely been studied in patients with BAV but further evidence is needed especially for patients with TAV, which is the dominating valve phenotype in aortic dilation.^10,11^

Circulating biomarkers represent another, more accessible, way to potentially improve risk stratification of aortic dilation. Interestingly, aneurysmal aortic tissue has been shown to secrete the multifaceted inflammatory cytokine interleukin-6 (IL-6) into the circulation.^12^ Promising results have also been reported for extracellular matrix (ECM) biomarkers such as matrix metalloproteinases (MMPs), tissue inhibitors of MMPs (TIMPs), and collagen fragments.^13–16^ Notwithstanding the promising initial findings on biomarkers of aortic dilation, mechanistic interpretations of the observed associations are unclear, and the effectiveness of biomarkers in the risk stratification of patients remains to be clarified.

Investigating associations between haemodynamics and circulating biomarkers could improve our understanding of aortic dilation mechanisms and aid in the identification of biomarkers linked to adverse haemodynamics. Interestingly, a recent study reported several correlations between WSS estimated with computer simulations and MMPs and presented a model for prediction of aortic surgery that combined these entities.^17^ Although other inflammatory and collagen turnover biomarkers were not investigated, these early findings highlight the potential of studies combining these two approaches.

The aim of this study was to explore associations between established haemodynamic markers measured with 4D flow CMR and circulating biomarkers in ascending aortic dilation patients with TAV.

## Methods

### Study population

The study population is a sub-cohort of the Swedish CArdioPulmonary bioImage Study (SCAPIS) in Linköping (*n* = 5058) and has been described previously.^11^ Coronary computed tomography (CT) angiography, chest CT, transthoracic echocardiography, blood pressure, anthropometrics, medical health records, and routine laboratory analyses were acquired within the SCAPIS study between 2015 and 2018.^18^ Ascending aortic diameter in the widest part of the ascending aorta was measured on the non-contrast CT images. Following guidelines for the diagnosis of aortic disease, subjects with an ascending aortic diameter ≥ 40 mm were identified as cases with dilation (*n* = 70).^2,11,19,20^ Additionally, age- and sex-matched subjects with ascending aortic diameter < 40 mm were recruited from SCAPIS as controls (*n* = 141). The study was approved by the Swedish Ethical Review Authority (2010-228-31 M and 2018/24–31). All study participants gave written informed consent.

### Circulating biomarkers

**Figure 1:**
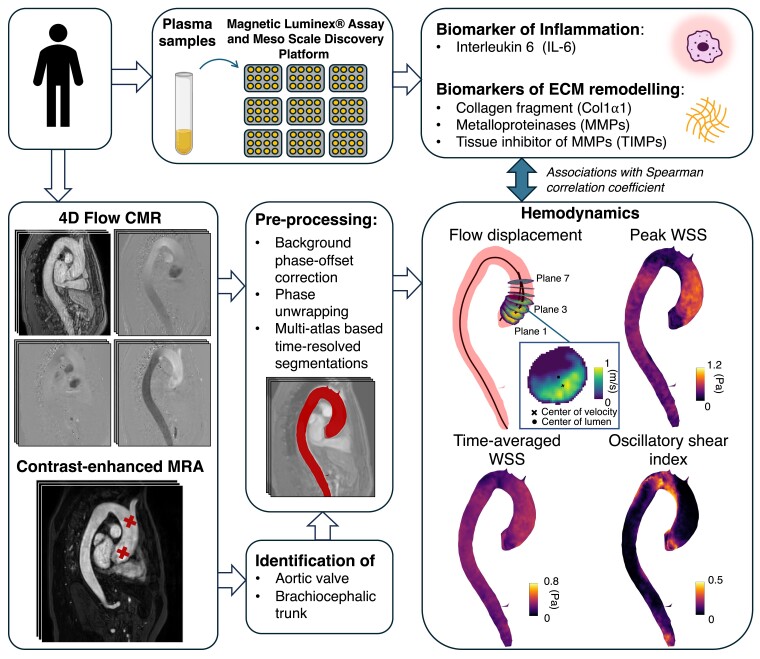
Overview of the methods: blood plasma samples were analysed to quantify biomarkers of inflammation and ECM remodelling; 4D flow CMR images were pre-process and flow displacement, WSS, TAWSS, and OSI were computed in the ascending aorta; WSS, wall shear stress.

Plasma samples were gathered from peripheral venous blood at the time of inclusion in SCAPIS and were stored at −80°C until analysis. Quantification of IL-6 was performed with the Meso Scale Discovery (MSD) platform (Rockville, MD, USA), an electrochemiluminescence assay. Circulating biomarkers of ECM remodelling [MMP-1, -2, -3, -9, -12; TIMP-1, -2, -3, -4, and type I collagen α1 chain (COL1α1)] were quantified with a commercially available Magnetic Luminex® Assay (R&D Systems Inc., Minneapolis, MN, USA) (*Figure 1*).^21^ Samples were read on the Bio-Plex® 200 platform with the Bio-Plex® manager software v.6.0 (Bio-Rad Laboratories Inc., Hercules, CA, USA). Internal control plasma duplicates were used on each assay plate to measure the inter-assay coefficient of variation (CV). The IL-6 assay had an inter-assay CV of 21%. The inter-assay CV was <10% for all ECM assays except for COL1α1 and MMP-9, which had an inter-assay CV of 33% and 17%, respectively.

### Haemodynamic markers

Following completion of the main SCAPIS study, haemodynamics was measured in the ascending aorta using 4D flow CMR at a 1.5T Philips Ingenia scanner (Philips Healthcare, Best, Netherlands) during 2020–21 (*Figure 1*). The 4D flow CMR data were acquired with a free-breathing, respiratory navigator-gated, retrospectively cardiac-gated gradient-echo sequence immediately after a contrast-enhanced magnetic resonance angiography (CE-MRA) with a Gadolinium-based contrast agent (Gadovist, Bayer Schering Pharma AG, Berlin, Germany). Scan parameters included a sagittal-oblique slab with a matrix size of 144–160 × 144 × 160–224 × 26–36, an isotropic acquired spatial resolution of 2.5 × 2.5 × 2.5 mm^3^, a velocity encoding range of ±150 cm/s, a flip angle of 10°, an echo time of 3.0 ms, a repetition time of 5.2 ms, a k-space segmentation factor of 2, and a SENSE parallel imaging acceleration factor of 2 × 1.6. Total scan time, including respiratory navigator gating, was 10–15 min. 4D flow datasets were corrected for concomitant gradient field effects on the scanner as well as phase wraps and background phase-offset errors offline.^22,23^

Time-resolved segmentations of the aorta were generated with a semi-automatic multi-atlas based method, which is described in more details in the Supplementary material.^24^ An example of a time-resolved segmentation is shown in the Supplementary data online, *Video S1*. The location of the aortic valve and brachiocephalic trunk were identified in the CE-MRA images and used to define the ascending aorta. Haemodynamic markers derived from 4D Flow included peak WSS, time-averaged WSS (TAWSS), and the oscillatory shear index (OSI) reported as mean and as maximum (99th percentile) in the ascending aorta. WSS was computed with established methodology.^25,26^ Peak WSS was defined as WSS at peak systole, and TAWSS as the average WSS magnitude over the cardiac cycle. The OSI is a measure of the degree to which WSS changes direction during the cardiac cycle and is used as a measure of disturbed WSS.^27,28^ Additionally, to further the understanding of OSI in aortic dilation, the relationship between mean OSI and the axial and circumferential components of mean WSS in the ascending aorta at peak systole were determined. Furthermore, normalized flow displacement was computed in seven equidistant planes along the ascending aorta as a measure of flow eccentricity.^29^ The average and maximum flow displacement in the seven planes were recorded.

### Statistical analysis

Descriptive statistics are presented as mean ± standard deviation for normally distributed variables and median (interquartile range) for non-normally distributed variables. The Kolmogorov–Smirnov test with an *α*-level of 0.05 was used to test if data were normally distributed. Student’s *t*-test and Pearson correlation were used for normally distributed variables, whereas Mann–Whitney *U* test and Spearman correlation were used for non-normally distributed variables. Fisher’s exact test was used to compare categorical variables between groups. A two-tailed *P*-value ≤ 0.05 was considered statistically significant. For the explorative correlation analyses between haemodynamics and circulating biomarkers, a Benjamini–Hochberg correction with a false discovery rate < 0.05 was applied to control for false discovery. Statistical analyses were performed in SPSS v.29 (IBM SPSS, New York, NY, USA).

## Results

### Subject characteristics

A total of 70 cases with aortic dilation were invited to undergo CMR and 58 consented to participate. Data from three cases were non-evaluable due to image quality issues (no contrast agent used, mislocated image volume, failure of the segmentation method). Seven cases with BAV and one case with a mechanical valve were excluded, resulting in a case group of 47 subjects with aortic dilation and TAV. An age- and sex-matched control group of 58 subjects with TAV was also invited to the CMR examination. Two subjects declined participation in the CMR study and the acquisition of CMR images was aborted in one. Data from four controls were non-evaluable due image quality issues (no contrast agent used, mislocated image volume). Further, blood samples from SCAPIS were missing for one control. This resulted in a control group of 50 subjects with TAV and aortic diameter < 40 mm. Demographics and clinical characteristics of the cases and controls are presented in *Table 1*. Cases had higher body mass index, higher diastolic blood pressure, and a higher prevalence of mild aortic regurgitation as assessed by echocardiography. None of the subjects appeared to have a genetic disorder associated with increased risk of aortic dilation, based on comprehensive questionnaires responded to by the subjects in SCAPIS.

**Table 1:** Baseline characteristics in the study population (*n* = 97)

Characteristics	Cases (*n* = 47)	Controls (*n* = 50)	*P*
Demographics			
Age (years)^a^	59.6 ± 3.91	58.9 ± 4.36	0.403
Women *n* (%)	6 (12.8)	9 (18.0)	0.579
Smokers *n* (%)	2 (4.3)	5 (10.0)	0.437
Anthropometrics			
BMI (kg/m^2^)^a^	27.9 ± 3.84	26.2 ± 4.11	**0**.**042**
BSA (m^2^)^a^	2.07 ± 0.18	1.98 ± 0.21	0.035
Blood pressure			
SBP (mmHg)^a^	136.1 ± 14.8	130.0 ± 17.0	0.062
DBP (mmHg)^a^	86.9 ± 8.65	81.4 ± 9.29	**0**.**003**
Comorbidities and medication			
Antihypertensive medication *n* (%)	17 (36.2)	13 (26.0)	0.380
Lipid-lowering medication *n* (%)	11 (23.4)	9 (18.0)	0.618
Diabetes *n* (%)	3 (6.4)	3 (6.0)	1.0
Aorta and aortic valve			
AR *n* (%)^b^	10 (21.7)	3 (6.1)	**0**.**037**
AV_max_ (m/s)	1.33 (1.2–1.5)	1.27 (1.15–1.47)	0.397
Aortic stenosis (%)	0 (0)	1 (2.1)	1.0
Aortic diameter (mm)^a^	42.8 ± 2.48	33.8 ± 2.8	**<0**.**001**
BSA-adjusted aortic diameter (mm)^a^	20.8 ± 2.48	17.2 ± 2.03	**<0**.**001**
Laboratory variables			
Haemoglobin (g/L)	151 (142–155)	148 (137.0–155.0)	0.388
Leucocytes (10^9^/L)	5.1 (4.4–6.1)	5.4 (4.8–6.6)	0.249
Thrombocytes (10^9^/L)^a^	236 ± 38	235 ± 62	0.877
HbA1c (mmol/mol)	34 (32–40)	35 (33–38)	0.589
Triglycerides (mmol/L)	1.20 (0.80–1.40)	0.97 (0.76–1.40)	0.232
Non-HDL cholesterol (mmol/L)^a^	3.78 ± 1.03	3.71 ± 0.87	0.799
Creatinine (μmol/L)^a^	82 ± 12	84 ± 11	0.408
eGFR_crea_ (mL/min/1.73 m^2^)^a^	77 ± 8.2	75 ± 7.1	0.241
hsCRP (mg/L)	0.9 (0.5–1.6)	1.2 (0.58–2.62)	0.362

Values presented as mean ± SD^a^ or median (IQR), and bold indicates statistical significance (*P* < 0.05 from the independent samples *t*-test^a^, Mann–Whitney *U* or Fischer exact test; all aortic regurgitations (^b^) considered mild except one case who had moderate regurgitation.

AR, aortic regurgitation; AV_max_, aortic valve maximum; BMI, body mass index; BSA, body surface area; CACS, coronary artery calcium score; DBP, diastolic blood pressure; SBP, systolic blood pressure; f-glucose, fasting glucose; HbA1c, haemoglobin A1c; HDL, high density lipoprotein; hsCRP, high sensitivity C-reactive protein; LDL, low density lipoprotein.

### Relationships between haemodynamics, biomarkers, and aortic diameter

**Figure 2:**
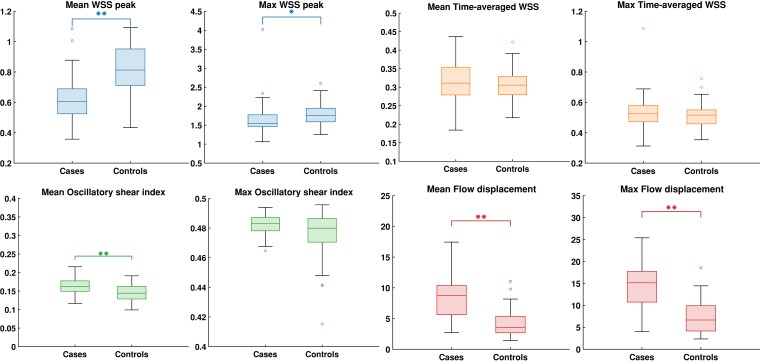
Distribution of haemodynamic parameters in dilated cases and controls represented as boxplots for mean and maximum values in the ascending aorta or at the dilated segment; *P < 0.05, **P < 0.001; WSS, wall shear stress.

**Figure 3:**
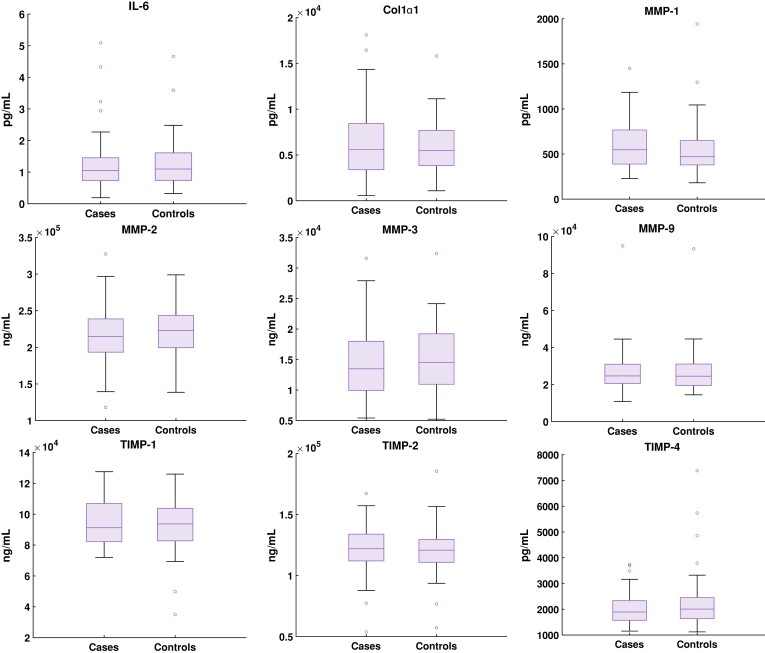
Distribution of circulating biomarkers levels in plasma in dilated cases and controls represented as boxplots; IL-6, interleukin-6; COL1α1, type I collagen α1 chain; MMP, matrix metalloproteinase; TIMP, tissue inhibitor of MMP.

Results on haemodynamics and biomarkers in cases and controls are shown as boxplots in *Figures 2* and *3*, for which the underlying numerical results are presented in Supplementary data online, *Tables S1* and *S2*, respectively. For haemodynamics, mean WSS (0.62 ± 0.15vs 0.82 ± 0.16, *P* < 0.001) and maximum WSS (1.54 (1.46–1.78) vs. 1.76 (1.59–1.95), *P* = 0.002) were lower, whereas mean flow displacement (8.72 (5.52–10.41) vs. 3.57 (2.67–5.39), *P* < 0.001), maximum flow displacement (15.14 (10.46–17.8) vs. 6.66 (4.14–10.0), *P* < 0.001) and mean OSI (0.16 ± 0.023 vs. 0.14 ± 0.022, *P* < 0.001) were higher in cases compared with controls. Among the circulating biomarkers, MMP-12 and TIMP-3 were excluded due to concentrations below the lower limit of quantification in most participants. None of the biomarkers differed between cases and controls. All haemodynamic markers that were different between cases and controls also correlated with ascending aortic diameter on CT, except for maximum WSS. TIMP-4 was the only biomarker that correlated to aortic diameter (*Table 2*). The axial component of mean WSS at peak systole was similar in cases and controls (0.18 [0.15–0.23] vs. 0.18 [0.15–0.22], *P* = 0.203), whereas circumferential peak WSS was higher in cases than in controls (0.73 [0.61–0.89] vs. 0.51 [0.46–0.65] *P* < 0.001). OSI correlated negatively with axial peak WSS (*r* = −0.289, *P* = 0.004) and positively with the circumferential component of peak WSS (*r* = 0.413, *P* = 0.002).

**Table 2:** Correlations (*r*, *P*) between ascending aortic diameter, BSA-adjusted ascending aortic diameter, haemodynamics, and biomarkers

Haemodynamic markers/biomarkers	Ascending aortic diameter^a^	BSA-adjusted ascending aortic diameter^a^
Max WSS	−0.188 (0.065)	−0.180 (0.078)
Mean WSS^a^	**−0.563 (<0.001)**	**−0.497 (<0.001)**
Max TAWSS	0.149 (0.145)	0.094 (0.359)
Mean TAWSS^a^	0.085 (0.410)	−0.065 (0.527)
Max OSI	0.123 (0.232)	0.004 (0.968)
Mean OSI^a^	**0.462 (<0.001)**	**0.294 (0.003)**
Max FD	**0.717 (<0.001)**	**0.647 (<0.001)**
Mean FD	**0.713 (<0.001)**	**0.649 (<0.001)**
IL-6	0.013 (0.899)	−0.004 (0.972)
COL1a1	0.031 (0.766)	0.100 (0.330)
MMP-1	0.059 (0.565)	0.102 (0.320)
MMP-2^a^	−0.110 (0.285)	−0.081 (0.429)
MMP-3^a^	−0.041 (0.688)	−0.067 (0.514)
MMP-9	−0.028 (0.785)	0.052 (0.615)
TIMP-1^a^	0.121 (0.239)	0.100 (0.330)
TIMP-2	0.006 (0.951)	0.030 (0.768)
TIMP-4	**−0.218 (0.032)**	−0.140 (0.171)

Pearson for normally distributed variables^a^ and Spearman correlation coefficients, where bold indicates statistical significance (*P* < 0.05).

COL1α1, type I collagen α1 chain; FD, flow displacement; IL-6, interleukin-6; MMP, matrix metalloproteinase; OSI, oscillatory shear index; TAWSS, time-average WSS; TIMP, tissue inhibitor of metalloproteinases; WSS, wall shear stress.

### Correlations between haemodynamics and biomarkers

**Figure 4:**
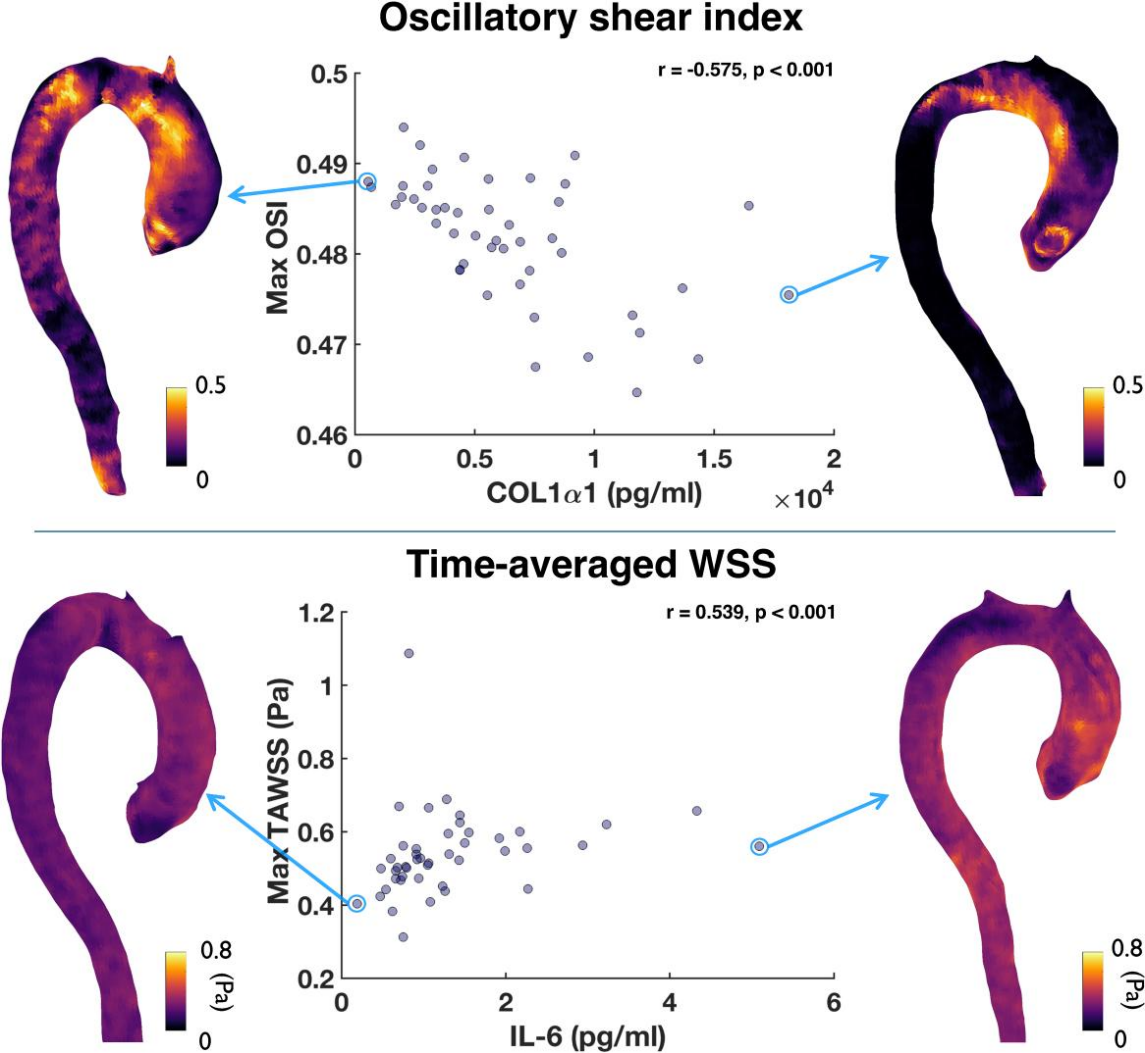
Scatter plots showing the distribution of maximum OSI vs. COL1α1 (top) and maximum TAWSS vs. IL-6 (bottom). The colormaps show distribution of OSI (top) and TAWSS (bottom) in the ascending aorta for the subjects with min/max COL1α1 (top) and IL-6 (bottom), highlighted with an arrow; *r*, Spearman correlation coefficient; OSI, oscillatory shear index; TAWSS, time-averaged wall shear stress; COL1α1, type I collagen α1 chain; IL-6, interleukin-6.

Correlations between haemodynamics and plasma biomarker levels are presented in *Table 3*. The associations that remained significant after Benjamini–Hochberg correction were a positive moderate correlation between IL-6 and maximum TAWSS in cases (*r* = 0.539, *P* < 0.001) and a negative moderate correlation between COL1α1 and maximum OSI in cases (*r* = −0.575, *P* < 0. 001) (*Figure 4*), and weak for the whole cohort (*r* = −0.348, *P* < 0. 001).

**Table 3:** Correlations between ascending aortic haemodynamics and plasma biomarker levels

Biomarker		Max WSS	Mean WSS^a^	Max TAWSS	Mean TAWSS^a^	Max OSI	Mean OSI^a^	Max FD	Mean FD
IL-6	All	0.127 (0.218)	0.091 (0.380)	**0.216 (0.035)**	0.189 (0.067)	0.015 (0.888)	0.072 (0.486)	0.023 (0.825)	0.021 (0.837)
	Cases	0.266 (0.078)	0.140 (0.359)	**0.539 (<0.001)** ^b^	**0.464 (0.001)**	0.200 (0.187)	0.074 (0.629)	0.126 (0.411)	0.069 (0.651)
	Controls	0.011 (0.937)	0.058 (0.690)	−0.070 (0.628)	−0.093 (0.522)	−0.151 (0.296)	0.082 (0.570)	0.008 (0.956)	0.004 (0.980)
COL1a1	All	−0.070 (0.498)	−0.088 (0.391)	−0.106 (0.301)	−0.071 (0.489)	**−0.348 (<0.001)** ^b^	−0.159 (0.119)	−0.009 (0.934)	0.026 (0.803)
	Cases	0.096 (0.520)	0.082 (0.585)	−0.084 (0.575)	−0.105 (0.482)	**−0.575 (<0.001)** ^b^	**−0.316 (0.031)**	0.013 (0.930)	0.110 (0.462)
	Controls	−0.259 (0.069)	**−0.281 (0.048)**	−0.142 (0.324)	−0.045 (0.757)	−0.161 (0.264)	−0.064 (0.658)	−0.080 (0.582)	−0.065 (0.653)
MMP-1	All	−0.011 (0.915)	0.007 (0.945)	0.143 (0.162)	**0.216 (0.034)**	0.087 (0.399)	0.081 (0.432)	0.019 (0.857)	0.023 (0.821)
	Cases	0.110 (0.460)	0.221 (0.135)	0.061 (0.682)	0.198 (0.182)	0.079 (0.600)	0.049 (0.742)	−0.021 (0.888)	−0.003 (0.985)
	Controls	−0.070 (0.629)	−0.052 (0.722)	0.209 (0.145)	0.258 (0.070)	0.093 (0.522)	0.069 (0.633)	−0.089 (0.538)	−0.072 (0.620)
MMP-2^a^	All	−0.084 (0.413)	−0.036 (0.725)	−0.047 (0.647)	−0.021 (0.837)	**−0.275 (0.006)**	−0.186 (0.068)	−0.142 (0.165)	−0.104 (0.313)
	Cases	−0.094 (0.532)	−0.042 (0.779)	−0.096 (0.521)	−0.046 (0.757)	**−0.409 (0.004)**	−0.251 (0.089)	−0.176 (0.236)	−0.076 (0.613)
	Controls	−0.164 (0.256)	−0.133 (0.357)	0.004 (0.977)	0.025 (0.861)	−0.170 (0.238)	−0.090 (0.536)	−0.095 (0.511)	−0.086 (0.555)
MMP-3^a^	All	**0.206 (0.043)**	**0.200 (0.050)**	0.002 (0.982)	0.005 (0.961)	0.034 (0.741)	0.036 (0.726)	−0.095 (0.356)	−0.093 (0.365)
	Cases	0.060 (0.686)	0.031 (0.836)	−0.020 (0.895)	0.057 (0.703)	0.269 (0.068)	−0.039 (0.794)	−0.071 (0.633)	−0.079 (0.600)
	Controls	**0.302 (0.033)**	**0.284 (0.045)**	0.052 (0.721)	−0.038 (0.796)	−0.104 (0.472)	0.217 (0.131)	−0.001 (0.995)	0.006 (0.968)
MMP-9	All	−0.046 (0.656)	0.031 (0.760)	−0.026 (0.797)	0.022 (0.830)	0.049 (0.631)	−0.032 (0.759)	0.065 (0.525)	0.076 (0.459)
	Cases	−0.114 (0.446)	−0.068 (0.651)	−0.077 (0.606)	−0.070 (0.638)	−0.148 (0.319)	−0.051 (0.736)	0.203 (0.172)	0.275 (0.061)
	Controls	0.042 (0.771)	0.171 (0.235)	0.000 (0.998)	0.074 (0.609)	0.161 (0.265)	−0.051 (0.727)	0.015 (0.916)	−0.001 (0.993)
TIMP-1^a^	All	−0.127 (0.214)	−0.082 (0.422)	−0.129 (0.209)	−0.098 (0.340)	−0.017 (0.870)	−0.103 (0.314)	−0.077 (0.451)	−0.061 (0.552)
	Cases	−0.177 (0.234)	−0.010 (0.948)	−0.044 (0.767)	−0.013 (0.931)	0.033 (0.826)	−0.113 (0.449)	−0.114 (0.447)	−0.050 (0.737)
	Controls	−0.104 (0.474)	−0.106 (0.465)	−0.224 (0.118)	−0.192 (0.182)	−0.054 (0.712)	−0.150 (0.300)	−0.079 (0.584)	−0.104 (0.471)
TIMP-2	All	−0.075 (0.462)	−0.012 (0.907)	−0.043 (0.673)	−0.012 (0.905)	**−0.281 (0.005)**	−0.138 (0.178)	−0.156 (0.127)	−0.105 (0.305)
	Cases	−0.198 (0.183)	−0.150 (0.316)	−0.118 (0.430)	−0.085 (0.569)	−0.235 (0.111)	−0.116 (0.436)	**−0.357 (0.014)**	−0.247 (0.094)
	Controls	−0.020 (0.890)	0.152 (0.291)	0.032 (0.828)	0.065 (0.655)	**−0.329 (0.020)**	−0.169 (0.242)	−0.102 (0.480)	−0.102 (0.483)
TIMP-4	All	0.051 (0.618)	0.105 (0.307)	0.152 (0.137)	0.040 (0.699)	−0.035 (0.737)	**−0.298 (0.003)**	−0.111 (0.279)	−0.121 (0.239)
	Cases	0.077 (0.609)	−0.010 (0.949)	0.169 (0.255)	0.061 (0.685)	−0.171 (0.251)	−0.106 (0.478)	−0.152 (0.308)	−0.105 (0.481)
	Controls	−0.012 (0.935)	0.101 (0.484)	0.166 (0.248)	0.039 (0.790)	0.085 (0.559)	**−0.399 (0.004)**	0.027 (0.852)	0.000 (0.998)

Spearman or Pearson (normally distributed variables^a^) correlation coefficient followed by *P*-value is presented for each correlation, where bold indicates unadjusted statistical significance (*P* < 0.05) and ^b^ indicates statistically significance after Benjamini–Hochberg correction.

COL1α1, type I collagen α1 chain; FD, flow displacement; IL-6, interleukin-6; MMP, matrix metalloproteinase; OSI, oscillatory shear index; TAWSS, time-average WSS; TIMP, tissue inhibitor of metalloproteinases; WSS, wall shear stress.

## Discussion

The central finding of our study is the association between haemodynamics quantified locally in the ascending aorta and circulating biomarkers assessed systemically. These correlations were more pronounced in the cases with dilation than in the control group. The observed differences between cases and controls in haemodynamic markers that are affected by increasing vessel size corroborate previous findings in patients with TAV, and do not offer new insights into aortic dilation.^8,9^ Despite the haemodynamic differences, biomarkers were similar in cases and controls, but may exhibit greater discriminatory power in cases with more advanced aortic dilation.^13,16^

Importantly, we observed that the haemodynamic markers that correlated most strongly against biomarkers did not correlate to diameter and differ from those that others have reported to correlate with growth.^8^ In this way, our findings highlight the potential use of 4D flow CMR in clinical care to reveal haemodynamics associated with pathological processes in the vessel wall in patients with TAV and mild-to-moderate aortic dilation. This way of integrating 4D flow CMR into routine clinical protocols may enable more precise risk stratification with individualized follow-up intervals as well as detection of disease progression determined by factors beyond diameter.

The observed moderate correlations between circulating biomarkers and WSS parameters accentuate the growing recognition that local haemodynamics in the ascending aorta, inflammation, and ECM dysregulation are interconnected factors in aortic dilation. WSS serves as a key indicator of flow-mediated effects on the aortic wall and previous studies have identified elevated WSS measured with 4D flow CMR in regions of ECM dysregulation in patients with aortic dilation and BAV.^7,30^ Our findings suggest a compelling link between flow-mediated ECM dysregulation and inflammation in the aortic wall, which is reflected by biomarker expression in the circulation.^12,14^

Elevated TAWSS may promote inflammation in the aortic wall and tissue leakage of IL-6, aligning with previous findings of elevated IL-6 levels downstream of thoracic aneurysmal tissue in dilated TAV.^12^ Macrophages and T cells are markedly increased in ascending aortic aneurysmal tissue in parallel with ECM degradation and apoptosis of media cells. These inflammatory cells are potential candidates for the source of increased IL-6.^31^ Although other cytokines, such as interferon-γ and interleukin-1β, have shown increased levels at both the mRNA and protein levels,^32^ they were not included in this study.

The degree of WSS directional changes throughout the cardiac cycle, described by OSI, may influence collagen synthesis in the dilated aortic wall. While OSI is low in stenotic flows with associated high systolic WSS,^27,28^ we found that OSI is elevated in aortic dilation with normally functioning TAVs and that elevated OSI correlates positively with circumferential WSS at systole. This suggests that OSI is lower in patients with circumferential fluid motion in systole, such as helical flow. COL1α1 makes up the type I collagen molecule that is essential not only for aortic structure but also for cell-cell interactions in the medial micro milieu. Notably, a COL1α1 gene polymorphism associated with decreased tissue expression of COL1α1 appears to increase the risk of thoracoabdominal aortic aneurysms with ensuing dissection and rupture in mice.^33^ Additionally, COL1α1 has been found to be decreased in ascending aortic aneurysm tissue samples and to correlate inversely with aortic diameter in a cohort of aortic dilation patients with diameters ranging from 2–7 cm.^34^ However, an increased RNA expression of COL1α1 in whole blood has been observed in aneurysm patients compared with controls, highlighting the complex dynamics in collagen remodelling during aneurysm development and the need for further studies.^14,35,36^

MMPs and TIMPs are the biomarker families most frequently studied in aortic dilation.^7,13,15,16^ In our study, they correlated less strongly with haemodynamics. It can be noted that Pasta *et al*.^17^ found a relationship between peak systolic WSS and MMP-1, TAWSS and TIMP-1, and TAWSS and MMP-2 using serum biomarkers and computer-simulated haemodynamics. We could not confirm those findings in our study, potentially due to differences in study population, where the study by Pasta *et al*. included BAV patients (30% of their cohort) and patients at more advanced stages of the disease where MMPs and TIMPs seem to be more clearly altered.^13,16^

Interestingly, the strongest associations between haemodynamics and biomarkers were seen for WSS parameters that encompass the whole-cardiac cycle. Elevated WSS at peak systole has been found in regions of wall ECM dysregulation characterized by elastin thinning and high levels of MMPs in BAV patients with large diameters.^7,30^ Although these studies by Guzzardi *et al*. and Bollache *et al*. did not report whole-cycle WSS parameters, the accumulated findings to date raise the possibility that while peak systolic WSS is a marker of wall dysregulation in BAV and stenotic patients, whole-cycle WSS parameters could be a marker of wall dysregulation in TAV patients.

### Study limitations

This study has several limitations. First, CMR was performed 4.4 ± 0.7 years after venous blood sampling and changes in haemodynamics and biomarkers could have occurred over this time. However, haemodynamics in aortic dilation and IL-6 levels in patients with high cardiovascular risk appear temporally stable over several years,^37–39^ and growth rate is generally slow in mildly dilated aortas.^1,3^ Secondly, our study comprised a limited number of study participants, including few women that hindered sex-stratified analysis. However, the size and composition of this cohort were a direct result of population-based screening of maximum ascending aortic diameter in ∼5000 middle-aged individuals. Additional studies are warranted to confirm the associations identified here. Thirdly, while the inter-assay CV was low for most biomarkers, it was over 30% for COL1α1. However, the samples for cases and controls were randomly distributed across multiple plates and we therefore do not expect systemic bias but rather decreased ability to detect associations. Finally, WSS values estimated with 4D flow CMR are sensitive to the spatial resolution.^40^ However, the same isotropic spatial resolution was used in all individuals, and therefore WSS values within and between subjects should be comparable.

## Conclusions

In a population-based cohort consisting of mild-to-moderate ascending aortic dilation cases with TAV and age- and sex-matched TAV controls, significant associations were discovered between 4D flow CMR derived whole-cardiac cycle WSS and circulating biomarkers representing inflammation and collagen synthesis. The strengths of the associations that were significant after Benjamini–Hochberg correction were moderate in the cases with dilation. These findings suggest an interplay between WSS and the processes of inflammation and extracellular matrix remodelling in early aortic dilation. Further studies are needed to confirm the findings reported here in both larger and more diverse cohorts with longitudinal follow-up.

## Supplementary data

Supplementary data are available at *European Heart Journal - Cardiovascular Imaging* online.

## Supplementary Material

jeae130_Supplementary_Data

## Data Availability

Data are available under reasonable request, as long as in compliance with Linköping University Hospital policy.
