# Supplementary material for: Wall shear stress measured with 4D flow CMR correlates with biomarkers of inflammation and collagen synthesis in mild-to-moderate ascending aortic dilation and tricuspid aortic valves
Source: Eur Heart J Cardiovasc Imaging. 2024 May 15;25(10):1384–93. doi: 10.1093/ehjci/jeae130 (PMC11441033; doi:10.1093/ehjci/jeae130)
Supplement: jeae130_Supplementary_Data [file jeae130_supplementary_data.zip › SupplementaryMaterial.docx]

**Method for generating time-resolved segmentations**

Time-resolved segmentations were generated by adapting a multi-atlas based method for aortic acquisitions (1). With this method, non-rigid diffeomorphic registration is used to compute the displacement field between the magnitude images of all timeframes (2). The displacement field is used to generate a phase-contrast magnetic resonance cardio angiography (PC-MRCA) as described by Bustamante et al (3). Compared to a standard phase-contrast magnetic resonance angiography (PC-MRA), the contrast between the aortic lumen and surrounding tissue is high both in systole and in diastole in the PC-MRCA.

In this study, five new atlases were created, using two cases and three controls. For each “atlas” subject, the aorta was manually segmented at the end-diastolic and end-systolic timeframes on the PC-MRCA using ITK-SNAP (4). These five atlases were used to automatically create segmentations for all the other subjects at the end-diastolic and end-systolic timeframes. The segmentations at these two timeframes were visually inspected for each subject, and manually adjusted with ITK-SNAP, when necessary. Two time-resolved segmentations were then obtained by registering in time the end-systolic and the end-diastolic segmentations, using the displacement field between the magnitude images at consecutive timeframes, as the described in (5). At each timeframe, the final segmentation is obtained as an average of the two registered time-resolved segmentation (the end systolic-based and end diastolic-based ones) weighted by the respective distance in the cardiac cycle, so that the systolic-based segmentation has higher weights in systolic timeframes, for example.

For computing wall shear stress, isosurfaces at each timeframe were created from the time-resolved segmentations. The isosurface at the end-systolic timeframe was registered then to the other those at the other timeframes, with an iterative closest point algorithm (6), to allow for computation of time-resolved parameters at each node on the isosurface representing the aortic wall. The moving contours were visually inspected for each subject, to ensure that they were properly following the ascending aorta and each timeframe.

To evaluate the impact of the method on the WSS analysis, a manual segmentation was created for the first 30 subjects (22 cases and 9 controls) that were acquired. The aorta was segmented manually on PC-MRA at the time frame with higher contrast in the lumen. WSS was computed with the same method. Percentage absolute differences between the multi-atlas based and the manual segmentations were 5.8 ± 4.5 and 5.6 ± 5.9 for mean and maximum WSS in the ascending aorta, respectively.

**References**

1. Bustamante M, Gupta V, Forsberg D, Carlhäll CJ, Engvall J, Ebbers T. Automated multi-atlas segmentation of cardiac 4D flow MRI. Med Image Anal. 2018 Oct;49:128–40.

2. Forsberg D. Robust Image Registration for Improved Clinical Efficiency : Using Local Structure Analysis and Model-Based Processing. 2013 [cited 2024 Mar 9]; Available from: https://urn.kb.se/resolve?urn=urn:nbn:se:liu:diva-91116

3. Bustamante M, Gupta V, Carlhäll CJ, Ebbers T. Improving visualization of 4D flow cardiovascular magnetic resonance with four-dimensional angiographic data: generation of a 4D phase-contrast magnetic resonance CardioAngiography (4D PC-MRCA). J Cardiovasc Magn Reson. 2017 Jun 23;19(1):47.

4. Yushkevich PA, Piven J, Hazlett HC, Smith RG, Ho S, Gee JC, et al. User-guided 3D active contour segmentation of anatomical structures: significantly improved efficiency and reliability. NeuroImage. 2006 Jul 1;31(3):1116–28.

5. Bustamante M, Petersson S, Eriksson J, Alehagen U, Dyverfeldt P, Carlhäll CJ, et al. Atlas-based analysis of 4D flow CMR: Automated vessel segmentation and flow quantification. J Cardiovasc Magn Reson. 2015 Dec;17(1):87.

6. Amberg B, Romdhani S, Vetter T. Optimal Step Nonrigid ICP Algorithms for Surface Registration. In: 2007 IEEE Conference on Computer Vision and Pattern Recognition. 2007. p. 1–8.

**Supplementary Tables**

| **Supplementary Table 1. Hemodynamics from 4D Flow CMR in Cases with Ascending Aortic Dilation and controls** | | | | |
| --- | --- | --- | --- | --- |
| **Hemodynamics** | **Median (IQR) or mean ± SD** | | | |
|  | **Total (n=97)** | **Cases (n=47)** | **Controls (n=50)** | ***p*** |
| Max WSS (Pa) | 1.66 (1.49-1.89) | 1.54 (1.46-1.78) | 1.76 (1.59-1.95) | **0.002** |
| Mean WSS (Pa) ^a^ | 0.72±0.18 | 0.62±0.15 | 0.82±0.16 | **<0.001** |
| Max TAWSS (Pa) | 0.52 (0.46-0.56) | 0.52 (0.47-0.58) | 0.52 (0.46-0.55) | 0.228 |
| Mean TAWSS (Pa) ^a^ | 0.31±0.049 | 0.32±0.054 | 0.31±0.04 | 0.443 |
| Max FD (%) | 10.28 (6.00-15.28) | 15.14 (10.46-17.8) | 6.66 (4.14-10.0) | **<0.001** |
| Mean FD (%) | 5.42 (3.15-8.95) | 8.72 (5.52-10.41) | 3.57 (2.67-5.39) | **<0.001** |
| Max OSI (-) | 0.48 (0.48-0.49) | 0.48 (0.48-0.49) | 0.48 (0.47-0.49) | 0.139 |
| Mean OSI (-) ^a^ | 0.15±0.024 | 0.16±0.023 | 0.14±0.022 | **<0.001** |
| Values presented as mean ± SD ^a^ or median (IQR). P-values calculated with independent samples t-test ^a^ or Mann-Whitney U analysis. Bold indicates statistical significance (*p* <0.05) between case and control group. 4D flow CMR = four-dimensional flow cardiac magnetic resonance imaging; FD = flow displacement; OSI = oscillatory shear index; TAWSS = time-average wall shear stress; WSS = wall shear stress | | | | |

| **Supplementary Table 2. Plasma Biomarker Levels in Cases with Ascending Aortic Dilation (>=40mm) and Controls (<40mm)** | | | | |
| --- | --- | --- | --- | --- |
| **Biomarker** | **Median (IQR) or mean ± SD** | | | |
|  | **Total (n=97)** | **Case (n=47)** | **Controls (n=50)** | ***p*** |
| IL-6 (pg/mL) | 1.060 (0.74-1.53) | 1.050 (0.73-1.47) | 1.10 (0.73-1.63) | 0.803 |
| COL1α1 (pg/mL) | 5570 (3700-7980) | 5592 (3390-8500) | 5474 (3830-7750) | 0.670 |
| MMP-1 (pg/mL) | 487 (382-734) | 548 (388-770) | 471 (374-668) | 0.310 |
| MMP-2 (ng/mL) ^a^ | 219.2±38.4 | 216.1±41.8 | 220.9±35.2 | 0.543 |
| MMP-3 (ng/mL) ^a^ | 14.5±5.4 | 13.8±5.5 | 15.1±5.3 | 0.249 |
| MMP-9 (ng/mL) | 24.65 (20.0-31.2) | 24.65 (20.4-31.3) | 24.51 (19.1-31.9) | 0.829 |
| TIMP-1 (ng/mL) ^a^ | 92.82±15.7 | 93.64±14.0 | 92.05±17.3 | 0.621 |
| TIMP-2 (ng/mL) | 121.4 (112-131) | 122.0 (112-134) | 120.8 (111-130) | 0.707 |
| TIMP-4 (pg/mL) | 1967 (1610-2380) | 1892 (1557-2347) | 2007 (1631-2470) | 0.309 |
| Values presented as mean ± SD ^a^ or median (IQR). P-values calculated with independent samples t-test ^a^ or Mann-whitney U test for comparison between case and control group. All biomarkers quantified in plasma with the Luminex® assay except IL-6 that was quantified with the Meso Scale Discovery platform.  COL1α1 = type I collagen α1 chainIL-6 = interleukin-6; MMP = matrix metalloproteinase; SD = standard deviation; TIMP = tissue inhibitor of metalloproteinases | | | | |
